# Clinical Cases and Incidents

**Published:** 1872-08-01

**Authors:** N. S. Davis


### Clinical Reports.

### CLINICAL CASES AND INCIDENTS.

BY N. S. DAVIS, M. D.

Every physician in active practice meets, now and then, cases of a unique character, requiring for successful treatment either unusual remedies or new modes of using ordinary ones. The special peculiarity of such cases may depend on some permanent idiosyncrasy of the patient, or the interposition of some temporary mental or physical condition that modifies, for the time being, the susceptibilities of the patient. They are pre-eminently the cases that baffle and annoy the inexperienced and the routine practitioner.

It may help some of our readers out of a dilemma, and aid in relieving their patients from suffering, to place before them the following clinical facts :

*Case* 1.—Mrs. B., a middle-aged woman, mother of a family, living in the south part of the West Division, was attacked severely with what is usually styled "bilious colic." She sent immediately for Dr. L., her family physician, who during three or four days exhausted his skill in vain for her relief. He tried successively anodynes, fomentations, cathartics, enemas, etc.; but the stomach of the patient soon became irritable, and everything taken was speedily rejected by vomiting, while enemas returned without any fecal discharge. In the meantime the abdomen had become very largely distended and tympanatic, tender to the touch ; the extremities cool ; pulse small, quick, and weak ; respiration irregular, and occasional sighing ; frequent retching and vomiting ; with great restlessness and prostration. At this stage in the progress of the case I met the attending physician in consultation. It was evident that the case had arrived at a critical stage, and must be relieved soon, or the patient would be lost.

As the attending physician had just commenced a new prescription, it was agreed that it should be continued for two or three hours longer, when, if no relief was obtained, an infusion of tobacco should be used as an enema. The specified time passed without any degree of relief to the patient, and Dr. L. had one drachm of chewing tobacco put into one pint of boiling water, and when cool enough, about one-half the quantity was injected into the rectum as an enema. Within twenty minutes the patient became free from all pain. The countenance turned pale, accompanied by a sense of faintness ; and there speedily followed a very copious evacuation of the bowels. The evacuation did not cease until the intestines were thoroughly emptied. She soon rallied from the sense of exhaustion, and improved rapidly for three or four days, when the intestinal obstruction again returned, with a renewal of pain, abdominal distension, quickness of pulse, and vomiting. The attending physician again administered the infusion of tobacco as an enema, which speedily induced very large fecal evacuations and entire relief to all the bad symptoms. This time the relief was permanent, and the patient fully recovered, and has remained well since.

It is more than thirty years since we gave the infusion of tobacco, the first time, for relieving dangerous obstruction of the bowels, produced by irregular contraction of the muscular coat of the intestines.

The disease had been induced, apparently, by eating a large quantity of popped corn, and walking about three miles, carrying a baby only two weeks old.

On the same night after the long walk, she was attacked with severe pain in the abdomen, which was followed by all the symptoms attribued to "bilious colic." A very intelligent physician in the neighborhood was called and attended the case faithfully for six days, but failed to afford the patient any relief. Being on a visit to my brother, who resided in the same neighborhood, we passed the house of the sick woman together on our way to the adjoining town. Being recognized by a member of the family, we were stopped and requested to see the patient. We learned that the attending physician had been in a short time previous, had informed the family that further treatment was useless, and had left to visit other patients several miles distant. We found the patient presenting a haggard expression of countenance; cool extremities; a small, quick and weak pulse; the abdomen more distended than before her confinement, very tympanitic, and tender to pressure; prompt rejection of everything she took, with frequent retching; and mind wandering. The condition seemed fully to justify the unfavorable prognosis which had been given by her physician. Although far from my own home, and without any medicine, it occurred to me that there was still a probability that the powerful relaxing effect of tobacco might relieve the intestinal contraction and procure evacuations in time to save the life of the patient. Finding some chewing tobacco in the house, what was thought to be about one drachm was put into a pint of water boiling hot. It was stirred up frequently until it was no more than milk warm; then one-half of the infusion was injected into the rectum as an enema. We stepped out to look about the farm a little and to while away the time until the result was obtained. In twenty or thirty minutes a messenger came running after us, saying the patient was dying. We hastened in, when we found the family gathered around the bed, the patient as pale as a corpse, breathing slow and still, pulse a mere thread but slower, and the mind like one in a trance. The condition of the breathing soon satisfied us that she was not dying, and in ten minutes more an active rumbling was heard in the abdomen, quickly followed by a copious evacuation from the bowels. Several evacuations occurred in the next few hours, and in the fœces was easily recognized a quantity of the popped corn, wholly undigested, that she had eaten the week previous. Soon after the evacuation of the bowels, she rallied from the sedative effects of the tobacco, her stomach ceased to be irritable, and she retained bland nourishment. During the next twenty-four hours, the bowels moved so frequently as to require small doses of morphia to allay the irritability of the mucous membrane, but she subsequently progressed steadily to complete recovery. From that time to the present we have occasionally resorted to tobacco enemas for the removal of obstinate intestinal obstruction, not dependent on invagination, and generally with success. It is, however, a remedy of extreme power, and should be used with corresponding caution.

*Case* 2. Mrs. M——, after exercising more than usual for two or three days, was attacked during the night with severe pain in the left iliac and hypogastric regions, accompanied by constipation. She had also had a miscarriage not more than three weeks previous. The pain was exacerbating and very severe, extremities cool, and pulse small and quick, but neither much tenderness nor distension of the abdomen. A physician was called during the night, who endeavored to allay the severe paroxysms of pain by suitable doses of morphine. This afforded partial relief for a few hours but was soon followed by secondary nausea, and the prompt rejection of everything taken into the stomach.

In the meantime the pain in the lower part of the abdomen and back remained unabated. Most of the succeeding day was spent in efforts to allay the pain by anodynes, and enemas were given for procuring evacuation of the bowels, but with no relief to the patient. At evening, about twenty hours after the commencement of the attack, I was requested to see her. Her countenance was dejected; skin cool; pulse small and quick; lower part of the abdomen rather full and tender to the touch; urine scanty, and its passage painful; no movement of the bowels; and constant nausea with restlessness. Being satisfied that the pain was dependent mostly on uterine irritation, and that no preparation of opium would be tolerated even by subcutaneous injection without perpetuating the nausea and vomiting, she was advised to desist from all remedies by the mouth, and to have an enema of hydrate of chloral 20 grs., tincture of belladonna 20 gtts., in half a teacupful of water, slightly warm; and to repeat the same in one, two or three hours, as the pain might indicate.

Fomentations had already been applied over the abdomen, and they were continued. On visiting the patient the following morning, we learned that the first enema of chloral and belladonna was retained, and in half an hour the patient was so far relieved that she slept quietly, and continued to do so for two hours or more. In about three hours she had a slight passage from the bowels made up in part of the enema, and some pain following it. Another enema of the same materials was given. This again quieted her so far as to procure rest for the remainder of the night. In the morning we found her pupils moderately dilated; mouth dry, and face suffused with redness, evidently from the effects of the belladonna in the enemas. In all other respects she was much better. She was advised to rest quiet, take a teaspoonful of nitrous ether in a little water every three hours, to increase the action of the kidneys, and if the abdominal pains returned, to use an enema containing only half the quantity of chloral and belladonna that was used before. She had felt no nausea since the first enema. During the next twenty-four hours she had three additional enemas of the smaller quantity directed; the effects of the belladonna on the pupils and throat gradually disappeared, and the patient recovered with but little additional medication.

*Case* 3. Mr. R——, a young man, who was just recovering from a severe attack of inflammation in the right iliac region, resulting in the formation of a large internal abscess, but which had been entirely healed for ten days, was attacked suddenly and severely with pain in the region of the segmoid flexure of the colon. He had rode out during the day and exercised more than at any time previous since the beginning of his sickness. In the middle of the night he began to have disturbance of the bowels, three evacuations occurring in quick succession, and leaving him with intense pain in the left iliac region. No further evacuations occurred, and though warm fomentations were applied externally and an anodyne taken internally, the pain continued without abatement and I was called early in the morning. Knowing from previous trials that any of the preparations of opium would be followed by secondary nausea and persistent vomiting, it was very desirable to avoid their use, and yet the severity of the pain without fever, rendered an efficient anodyne influence highly important. He was directed to have an enema of slightly warm water, half a teacupful, containing twenty grains of hydrate of chloral and twenty pwts. of tincture of belladonna; and if not relieved in one hour, to repeat the same with the addition of twenty drops of the tincture of opium. The first enema not affording much immediate relief, took the second as directed, which relieved the pain so fully that he slept some and rested well for twenty-four hours. Fearing that the attack might result in inflammation and suppuration similar to what had occurred in the opposite side of the abdomen, he was kept at rest for several days. No bad symptoms followed, however, and he needed but little further treatment.

---

Dr. R. W. Taylor has been appointed Clinical Professor of Diseases of the Skin at the Woman's Medical College, New York.

